# Why sprint interval training is inappropriate for a largely sedentary population

**DOI:** 10.3389/fpsyg.2014.01505

**Published:** 2014-12-23

**Authors:** Sarah J. Hardcastle, Hannah Ray, Louisa Beale, Martin S. Hagger

**Affiliations:** ^1^Health Psychology and Behavioural Medicine Research Group, School of Psychology and Speech Pathology, Faculty of Health Sciences, Curtin UniversityPerth, WA, Australia; ^2^School of Sport and Service Management, University of BrightonEastbourne, UK

**Keywords:** exercise psychology, sprint interval training, exercise intensity, behavior-change, feeling states, exercise adherence

Public health practitioners and researchers in behavioral medicine recognize the need to find effective physical activity interventions and prescriptions to curb the growth in inactivity and prevent chronic illness (Conn et al., 2009; Hagger, 2010; Hardcastle et al., 2012; Katzmarzyk and Lear, 2012). For example, researchers in exercise physiology have focused on the minimal dose of exercise needed to gain favorable physiological adaptations to cardiovascular and metabolic systems (Gibala et al., 2012). Efforts to identify a minimal dose of exercise are linked to the problem of exercise adherence with few people meeting current physical activity guidelines of 30 min per day of moderate intensity exercise. Given that time is the most commonly cited barrier to exercise (Trost et al., 2002; Sequeira et al., 2011), exercise professionals have focused attention on the development of time-efficient exercise interventions (Gibala, 2007). A recent development is the advocacy of Sprint Interval Training (SIT) as a means to attain substantial health benefits with a lower overall exercise volume. SIT is characterized by repeated, brief (4–6 × <30 s), intermittent bursts of all-out exercise, interspersed by periods (approximately 4.5 min) of active or passive recovery (Gibala et al., 2012). Research has consistently demonstrated that participation in SIT results in a host of physiological adaptations including improvements in health and fitness indicators (Burgomaster et al., 2006, 2008; Gibala et al., 2006, 2012; Rossow et al., 2010; Tong et al., 2011). In addition, these improvements have been reported to be equal or superior to traditional continuous aerobic training despite SIT involving a substantially lower total overall training volume (Rossow et al., 2010; Tong et al., 2011; Gibala et al., 2012; Cocks et al., 2013). Consequently, SIT is being advocated as a time-efficient alternative intervention for the achievement of fitness and health benefits through exercise (Gibala, 2007; Whyte et al., 2013).

In this article we contend that SIT is unlikely to be taken up by the majority of the sedentary population and caution is needed before such training is advocated to the general public. Proponents of SIT have focused almost exclusively on physiological adaptations. However, the exclusive focus fails to consider whether a largely sedentary population will feel physically capable and sufficiently motivated to take up and maintain a regime of highly intense exercise. Based on theory and research in exercise psychology, we contend that the prospect of participating in SIT for previously sedentary individuals is likely to be considered too arduous and may evoke anticipated perceived incompetence, lower self-esteem, and potential failure (Williams and Gill, 1995; Hein and Hagger, 2007; Lindwall et al., 2011). They may likely be more inclined to avoid participating as a consequence. We also contend that should previously sedentary individuals be introduced to high intensity exercise of the type proposed in SIT it will likely evoke a high degree of negative affect that may lead to an avoidant response with the prospect of future sessions. In addition, we contend that SIT is a complex and structured regime that requires high levels of self-discipline and self-regulation and is, therefore, unlikely to be adopted outside the laboratory environment (Hagger, 2013; Hagger and Luszczynska, 2014). Finally, we debate the notion that SIT is time-efficient and suggest that it does not sufficiently address “lack of time” as a commonly-cited barrier to exercise (Hardcastle and Hagger, 2011).

In a largely sedentary population, the strenuous nature of SIT is likely to be a deterrent to participation because individuals tend to avoid exercise if they find it aversive. Several theories including social cognitive theory (Bandura, 1977), achievement motivation theory (Weiner, 1985) and self-determination theory (Deci and Ryan, 1985; Hagger et al., 2006; Chatzisarantis et al., 2007) contend that a high level of motivation and competence are needed to participate in regular physical activity. Typically, sedentary and low-active individuals do not feel competent in the physical domain and may not, therefore, feel sufficiently confident to engage in the activity (Teixeira et al., 2012). The motivation and effort required to participate in high intensity exercise is much higher than that needed to undertake activities of a moderate intensity (e.g. walking) (Williams and Gill, 1995; Tritter et al., 2013). If individuals feel unable to demonstrate competence in SIT, they are more likely to invest little effort in a prescribed activity or avoid it all together. Low competence, self-esteem, and motivation among sedentary individuals are a considerable problem for exercise promoters presented with the task of developing means to promote increased activity to a resistant population (Williams and Gill, 1995; Hein and Hagger, 2007).

In addition to competence and motivation, enjoyment is also a predictor of exercise adherence and most people do not enjoy high intensity exercise (Parfitt and Hughes, 2009). SIT may be inappropriate for a largely sedentary population because the negative affect that such supra-threshold intensities evoke could diminish intrinsic motivation and discourage exercise adherence. There is considerable evidence that adherence to exercise is influenced by affective responses to exercise intensity. In particular, enjoyment and feelings of pleasure have been shown to decrease as exercise intensity increases (Ekkekakis et al., 2011). The American College of Sports Medicine (ACSM) exercise guidelines state that exercise-induced feelings of fatigue and negative affect can act as a deterrent to continued participation (American College of Sports Medicine, 2013). The supra-threshold intensities induce a psychobiological stress response that is felt as unpleasant. Conversely, exercise intensities below these thresholds can be effective for improving health and fitness and are generally rated as more pleasant, and more likely to be tolerated by, most individuals irrespective of age or physical condition. In a study on sedentary adults' adherence to exercise prescriptions, Perri et al. (2002) found significantly greater adherence in the moderate intensity condition compared to the high intensity condition. Prescribing a higher frequency (5–7 days vs. 3–4) of exercise sessions increased the accumulation of exercise without a decline in adherence, whereas prescribing a higher intensity decreased adherence and resulted in the completion of less exercise over the 6 months. Although some studies have found SIT to be more enjoyable than continuous exercise (e.g., Bartlett et al., 2011), they have tended to focus on “recreationally active” participants meaning that the findings cannot be directly applied to an unfit and sedentary population. In addition, recent research has focused on strategies to reduce the negative affect experienced by individuals when performing SIT such as listening to music or receiving feedback to boost self-efficacy (e.g., Tritter et al., 2013; Stork et al., 2014). However, we contend that such endeavors are futile given that such types of training are unlikely to be adopted or maintained by sedentary individuals in the first place.

SIT is also inappropriate for a largely sedentary population because it is a relatively complex and structured exercise regime that requires a high degree of self-regulation to be effective. Mostly SIT protocols have been undertaken in laboratory settings under the supervision of exercise physiologists (e.g., Gibala et al., 2006; Burgomaster et al., 2008; Cocks et al., 2013; Whyte et al., 2013) not to mention the “significant encouragement provided during the Wingate tests” by the research team (Cocks et al., 2013, p. 645). The transfer of SIT to an unsupervised setting in which the onus is placed on inexperienced sedentary individuals to self-select the appropriate intensity is likely to be problematic. Individuals will need to know the speed and effort required to work during the supra-maximal and active recovery periods. They will also require self-monitoring tools at hand such as a stopwatch to time the intervals, and will also need to have sufficient know-how to undertake an appropriate warm up and cool down to prevent injury. It is unlikely that sedentary individuals with low levels of experience with exercise are going to be able to be independently successful in a complicated activity like SIT. Further research is needed to explore whether the SIT can be successfully implemented and maintained in a real life setting with those insufficiently active and unfamiliar with vigorous intensity exercise.

Finally, we question the often-cited benefit of SIT that it is time-efficient. If the minimal number of intermittent bursts of activity is four followed by four 4.5 min breaks, then at least 20 min is needed and this does not include a warm up or cool down. Therefore, in reality individuals would still need to free up 30 min in order to participate, even if on fewer days of the week (three as opposed to five). For sedentary individuals, a time-efficient means to engage in a programme of regular physical activity and structure it around a busy lifestyle is attractive. However, coupled with other concerns about the adverse psychological effects of high-intensity exercise regimens like SIT, and their relative complexity to undertake, we contend that lower intensity bouts of exercise of a similar duration are likely to be more appealing to sedentary individuals. Exercise of this nature may, therefore, be optimally effective in promoting adherence to exercise in this population.

In summary, we contend that although SIT appears to be an effective exercise modality for physiological benefit, it is unlikely to be effective as a means to promote regular participation in physical activity in a largely sedentary population. We have argued that SIT is inappropriate for sedentary individuals because engaging in such training requires high levels of motivation and confidence. In addition, high intensity exercise is likely to evoke to negative affect which may lead to subsequent avoidance of further exercise. SIT programmes of exercise are also relatively complex and involve a high degree of self-regulation, which may also be a barrier to continuation in those who are uninitiated. Finally, we contend that SIT should not necessarily be considered time efficient as a session would likely last at least 30 min. We would like to see further research that addresses the motivational factors and responses of sedentary people to SIT. Specifically, we propose the following research agenda to improve knowledge of SIT in sedentary populations: (1) the acceptability of, and affective responses to, SIT programmes; (2) the social cognitive and motivational factors that may be related to participation in, and adherence to, SIT programmes; (3) the degree of adherence to SIT programmes compared to programmes of continuous aerobic exercise of moderate intensity; and (4) whether SIT programmes can be transferred to natural settings outside of the supervised laboratory environment.

## Author contributions

Sarah J. Hardcastle conceived the ideas presented in the article and took the lead role in drafting the article. Hannah Ray, Louisa Beale, and Martin S. Hagger assisted in drafting the article.

### Conflict of interest statement

The authors declare that the research was conducted in the absence of any commercial or financial relationships that could be construed as a potential conflict of interest.
